# The Association of Abuse and Depression With Suicidal Ideation in Chinese Adolescents: A Network Analysis

**DOI:** 10.3389/fpsyt.2022.853951

**Published:** 2022-03-28

**Authors:** Kuiliang Li, Xiaoqing Zhan, Lei Ren, Nan Liu, Lei Zhang, Ling Li, Ting Chen, Zhengzhi Feng, Xi Luo

**Affiliations:** ^1^Department of Medical English, School of Basic Medical Sciences, Army Medical University, Chongqing, China; ^2^School of Psychology, Army Medical University, Chongqing, China; ^3^Department of Military Medical Psychology, Fourth Military Medical University, Xi’an, China; ^4^Department of Health Examination, People’s Hospital of Wansheng Economic Development Zone, Chongqing, China; ^5^College of General Education, Chong Qing Water Resources and Electric Engineering College, Chongqing, China

**Keywords:** adolescents, depression, child abuse, suicide ideation, network analysis

## Abstract

**Background:**

Abuse experiences in adolescents are associated with the risk of depression and suicide. Thus, there is an urgent need to develop prevention and intervention measures for clinicians, policymakers, and researchers.

**Methods:**

Network analysis method was used to analyze the cross-sectional data of Chinese adolescents in this study. The Patient Health Questionnaire for Adolescents (PHQ-A) was used for assessing depression, in which item 9 of the PHQ-A was used to assess suicide ideation, and International Society for the Prevention of Child Abuse and Neglect (ISPCAN) Child Abuse Screening Tool-Children’s Home Version (ICAST-CH) was used for assessing abuse.

**Results:**

The prevalence of suicidal ideation among Chinese adolescents was 21.46% (95% CI, 20.79–22.16%). The prevalence of moderate or severe depression was 16.76%, and the prevalence of violence exposure, psychological victimization, neglect, and physical victimization was 33.5%, 59.5%, 28.37%, and 31.51% in the past years, respectively. Network analysis results showed that the most central nodes in the network of abuse and depression were “unimportant,” “not cared,” and “pushed.” The bridge nodes were “suicidal ideation” and “unimportant.” The nodes “sadness,” “failure,” and “unimportant” explained the largest proportion of the variance of suicidal ideation in our network. Differences were found in the structure of both abuse and depression networks between adolescents with or without suicidal ideation.

**Limitations:**

The self-reporting–based cross-sectional surveys and community sample groups limit the inference of causality and the generalization of the results.

**Conclusion:**

This study shows that “unimportant” is the central and bridge nodes in the abuse and depression networks and also explains a part of variance of suicidal ideation. The effect of “unimportant” should be considered in the prevention and intervention of depression and suicide in adolescents with abuse experience. Future study is needed to confirm its role in clinical intervention.

## Introduction

Over 264 million people of different ages suffer from depression globally, which is a leading cause of disability and a major contributor to the global burden of disease (1). Depressive symptoms include changes in moods and psychological behaviors (2), and major depression can contribute to the development of suicidality or affect social functions (3, 4). Depression typically starts in childhood or adolescence, and its prevalence significantly increases in adolescence and slows down and stabilizes till the end of adolescence (5, 6). However, its recurrence rate is high in adulthood (7, 8). Many patients with depression do not receive effective treatment; previous research reported that only 19.5% of patients with major depression receive treatment in China (9). As the main depressive symptoms of adolescents may be quite different from those of adults (7), it is necessary to explore the characteristics of depressive symptoms of adolescents to guide early detection and intervention.

One major risk factor of depression is the experience of abuse, which widely exists in people of all ages, ranging from children to the elderly (10, 11). Compared with adults, there is a higher rate of abuse among children, while they are less resilient to it. Although the rate of child abuse in China decreased from 70.1% (12) in 2010 to 42.3–62.7% in 2019 (13), it is still much higher than that in Western developed countries (23.5–37.4%) (14, 15). Child abuse will not only lead to physical injury but also cause serious psychological trauma and even affect the mental health in adulthood (13, 16). It is reported that childhood abuse is a common cause of depression (17–19) and even increases the risk of depression and anxiety in adulthood (20).

The previous study reported that parents are responsible for 81% of child abuse, among whom 88% are biological parents (21), which means that family is the primary place where child abuse occurs. This phenomenon may be accounted by the strict education (the so-called “education through stick and club”) that is prevalent in China, which means that children will be punished if they are considered not behaving themselves, such as failure in examination, offending guardians, and not being obedient. A previous study has shown that frequent exposure to family abuse is an independent risk factor for depression in adulthood (22). Therefore, research on child abuse in families is helpful and necessary to understand the development of depressive symptoms. Emotional abuse and neglect in children are usually a risk factor of early onset of depression (23). Therefore, the effect of parental psychological abuse on children should be addressed to reduce the risk of depression in adolescents (24). In addition, abuse experience also affects the recovery from depression. Research shows that, during 12 months, the recovery rate of severely depressed women without abuse experience is 3.7 times higher than that of those with abuse experience (25).

Suicide is the second leading cause of death among adolescents (26), and the development of suicidality may be the most serious consequence of depression. The factors contributing to adolescent suicide include psychological factors (such as depression and anxiety), stressful life events (such as family problems and peer conflicts), and personality traits (27). The relationship of depression and anxiety symptoms with suicidal ideation has been explored in our previous study (28). However, depression and anxiety cannot fully explain suicidal ideation; other suicide risk factors are equally important, such as child abuse, which is reported to be directly associated with suicide. A total of 80% of people who attempt suicide have a history of child abuse (29), and abuse increases the risk of suicidal ideation and suicidality (30). A study in Hong Kong, China, shows that child abuse is an important risk factor for attempted suicide, 4.1% of children are hospitalized because of attempted suicide within 5 years after abuse, and the rate of suicide attempt rises to 7.1% within 20 years (31). This evidence shows that child abuse is an important risk factor for suicide. It is necessary to further investigate which type of abuse events has a direct effect on suicide to provide guidance for reducing the risk of suicide.

Network analysis provides a new perspective to understand the relationship between symptoms of psychological disorders and other variables of interest. Many previous studies have explored the relationship between depressive symptoms and other variables through network analysis, such as PTSD (32, 33), eating disorders (34), and negative life events (35). In addition, studies have explored the relationship between depressive symptoms and suicidal ideation (36). These studies provide insights into the relationship between depressive symptoms and suicidal ideation. However, so far, network analysis has not yet been conducted to investigate the relationship between abuse, depression, and suicidal ideation. Therefore, this study adopts network analysis to explore the network structure of all items of Patient Health Questionnaire for Adolescents (PHQ-A) and International Society for the Prevention of Child Abuse and Neglect (ISPCAN) Child Abuse Screening Tool-Children’s Home Version (ICAST-CH) in a large sample (*n* = 13754) of Chinese adolescents, as well as the relationship of abuse events and depressive symptoms with suicidal ideation. It also compares the differences in network structures between adolescents with or without suicidal ideation. The present study aims to identify the association between abuse events and depressive symptoms in adolescents, the potential targets for abuse and depression interventions and different targets of intervention between adolescents with or without suicidal ideation, so as to alleviate depression and reduce suicide risk.

## Materials and Methods

### Ethics Statement

This study has been reviewed and approved by the Medical Ethics Committee of the Department of Medical Psychology, Army Medical University (No. CWS20J007). The written approval was obtained from the officials of the sampled schools. The parents or legal guardians provided consent to participate in the study on behalf of the children. Participants read the informed consent before participating in this study and were reminded that the survey was anonymous and personal information would not be disclosed.

### Participants

**FIGURE 1:**
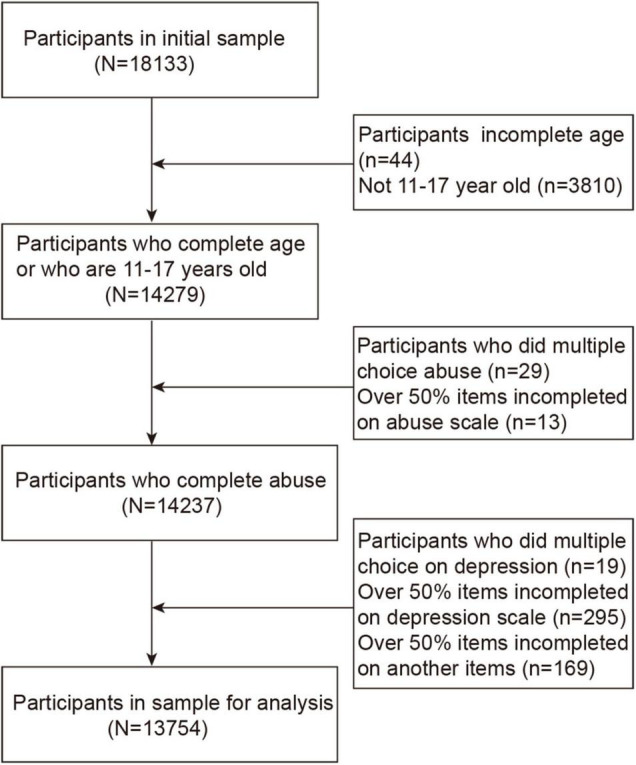
Flow of participants in the study.

This was a cross-sectional cluster sample survey that was conducted between October and November in 2020, which investigated 18,133 students from 32 schools in Chongqing, China, ranging from the third grade of primary school to the third grade of secondary school. By excluding 3,854 questionnaires of those younger than 11 and older than 17 years old and 525 ones with redundant choices or completion rates lower than 50%, we included the remaining 13,754 valid questionnaires in the final analysis (response rate, 75.85%). Data analysis was performed from March 2021 to June 2021. The sample flow diagram is shown in Figure 1.

### Measures

#### Symptoms of Depression

The depressive symptoms of adolescents were assessed by PHQ-A, which is the most commonly used screening tool for depressive symptoms (37). It includes nine items, such as “little interest or pleasure in doing things” and “feeling down, depressed, or hopeless.” The participants were asked to choose the frequency of each symptom in the last 2 weeks. The score of each item ranged from 0 (“not at all”) to 3 (“nearly every day”). The higher the total score is, the more severe the depression would be. The Cronbach’s α of PHQ-A in the current study was 0.893.

#### Suicide Ideation

Item 9 of the PHQ-A was used to assess suicide ideation: “thoughts that you would be better off dead, or of hurting yourself in some way?” The item is rated on a four-point scale: 0 = not at all; 1 = several days; 2 = more than half the days; and 3 = nearly every day. Adolescents who scored ≥ 1 were considered to meet the criteria for suicide ideation.

#### Events of Child Abuse in Families

Child abuse screening tool-children’s home was used to assess the abuse experience of the adolescents in the last year (38). The tool contains 36 items with five dimensions: violence exposure (seven items), emotional abuse (eight items), neglect (six items), physical abuse (nine items), and sexual abuse (six items). Because sexual abuse survey failed to pass the ethical review, it was excluded in the current study. The final survey included the remaining 30 items. Participants were asked to score each item (0–6 points) according to their experience in the last year (0: never happened, 1: happened but not in the last year, 2: one to two times, 3: three to five times, 4: six to 12 times, 5: 13 to 50 times, and 6: more than 50 times). Score 0 represents the absence of abuse and score 1–6 abuse of various levels. The total score ranges from 0 to 180. A high score indicates a high frequency of abuse or more types of abuse. Cronbach’s α of the scale was 0.904 in the current study.

### Data Analysis

#### Data Preparation

The data were imported into the Excel to exclude the questionnaires with redundant choices, and those with a response rate of less than 50% were also excluded. We imputed the remaining data using the mice package in R 4.0.0. The potential redundant nodes in the network were identified using the “goldbrick” function of the R *networktools* package. As a result, none of the items were suggested to be excluded in further analysis.

#### Graphical Least Absolute Shrinkage and Selection Operator Network

A network was constructed to explore the relationship between abuse events and depressive symptoms. The EBICglasso function of the R *qgraph* package was used to estimate the network (39). Graphical Least Absolute Shrinkage and Selection Operator (LASSO) algorithm was performed for regularization of Gaussian graphical model (GGM), which can shrink all edges and set the edges with small partial correlation to zero to obtain a more stable and easier to interpret network. The GGM is undirected, with its edges representing the partial correlation between the nodes in the network. In the visual network, the red edge represents the negative correlation between nodes, whereas the blue edge represents the positive correlation between nodes. A thicker edge indicates a stronger link between nodes.

#### Network Centrality, Predictability, and Robustness

The expected influence centrality index was calculated by the R *qgraph* package (40). A previous study has shown that expected influence is more appropriate for the network with both negative and positive edges when compared with the traditional node centrality index (e.g., strength centrality) (41). Thus, we chose expected influence as the centrality index in the current study. Expected influence is the sum of the value (e.g., correlation coefficients) of all edges connecting to a given node. The greater the expected influence of the node is, the more important it would be in the network. Moreover, we computed the predictability for each node *via* R *mgm* package (42). Predictability means the extent to which the variance of a node can be explained by all of its adjacent nodes.

The stability and accuracy of the network were calculated by the R *bootnet* package (43). First, the bootstrap approach (nBoots = 2,000) was used to evaluate the accuracy of edge weights *via* computing the 95% CI. The narrower the CI was, the more accurate the estimation of edge weight would be. Second, the case-dropping bootstrap method was used to evaluate the stability of the centrality index *via* calculating the correlation stability coefficient. The correlation stability coefficient should not be lower than 0.25, preferably higher than 0.50 (43).

#### Bridge Nodes

To identify important nodes that bridge the abuse-depression connection, we calculated the bridge expected influence (i.e., the sum of edge weights from a given node to the other community) *via* the R *networktools* package (44). From the perspective of statistics, nodes with higher bridge expected influence in one community have wider and closer connections with nodes in the other community. Thus, nodes with higher bridge expected influence are considered to play more central roles in activating nodes from the opposite community. In this study, all nodes were divided into two communities: one community contained 30 abuse events and the other one contained nine depressive symptoms.

#### Flow Network Analysis of Suicide Ideation

The “flow” function of the R *qgraph* package was used to layout the nodes directly or indirectly correlated to suicidal ideation. This layout placed “suicide ideation” on the left side as the target variable and constructed a horizontal network. Nodes in the network were directly or indirectly correlated with suicidal ideation by edges.

#### Network Comparison of Suicide Ideation

Finally, we used the *NetworkComparisonTest* package to compare whether differences existed in network structure and overall network connectivity between adolescents with or without suicidal ideation (permutations = 2,000) (45). We analyzed the global strength value and p-value of each network and the differences in network structure and the number of edges with significant differences.

## Results

### Descriptive Statistics

The sample used in this study consisted of 13,754 adolescents, aged from 11 to 17 years (*M* = 13.57, SD = 1.86). Of them, 6,806 were boys (49.48%), 6,880 were girls (50.02%), and 68 did not select a gender. The mean score of depression was 5.08 (SD = 5.30), and 16.76% of the participants had moderate or severe depressive disorder (see Table 1). The mean score of abuse was 24.30 (SD = 27.39), and 67.78% of the participants were abused at least once in the past year, whereas 85.55% were abused at least once in their lifetime, with psychological abuse being the most frequent (see Table 1). Abuse experiences were significantly associated with depression (Supplementary Table 1). Tables 2, 3 show the average, SD, and prevalence of the symptom and predictability of each item. Depression symptoms “anhedonia” and “fatigue” were the most frequent symptoms. Among the abuse events, “screaming” had the highest frequency, whereas “burned” had the lowest frequency. A total of 21.46% (95% CI, 20.79–22.16%) participants (*n* = 2,952) had suicidal ideation.

**TABLE 1:** Demographics, depressive severity and abuse rate of the participants (*n* = 13754).

	M (S.D.)/*n* (%)	
Age	13.57 (*1.86*)	
**Gender**		
Male	6806 (49.48)	
Female	6880 (50.02)	
Missed	68 (0.50)	
**Depression Severity (Total raw score)**		
0–4	7,904 (54.47)	
5–9	3,545 (25.77)	
10–14	1,344 (9.77)	
15–19	613 (4.46)	
20–27	113 (2.53)	

**Prevalence of abuse in past year and lifetime**	**Past year**	**Lifetime**

Violence exposure	4,608 (33.50)	8,637 (62.80)
Psychological victimization	8,184 (59.50)	10,576 (76.89)
Neglect	3,902 (28.37)	5,518 (40.12)
Physical victimization	4,334 (31.51)	8,814 (64.08)
Any abuse	9,322 (67.78)	11,767 (85.55)

*The SD in italics to distinguish them from %s in the other brackets.*

**TABLE 2:** Abbreviation, Mean, Standard Deviation, and Presence of PHQ-A Symptoms.

Depression symptoms	Abbreviation	Mean	SD	%Presence	Predictability
Feeling down, depressed, irritable, or hopeless	D1: Sadness	0.610	0.770	46.82	0.538
Little interest or pleasure in doing things	D2: Anhedonia	0.706	0.817	52.44	0.431
Trouble falling asleep, staying asleep, or sleeping too much	D3: Sleeping	0.570	0.854	38.44	0.467
Poor appetite, weight loss, or overeating	D4: Appetite	0.424	0.748	30.21	0.371
Feeling tired, or having little energy	D5: Fatigue	0.743	0.867	52.46	0.482
Feeling bad about yourself, or that you’re a failure or that you’ve let yourself or your family down	D6: Failure	0.748	0.918	49.73	0.548
Trouble concentrating on things like school work, reading, or watching TV	D7: Concentration	0.675	0.889	45.45	0.438
Moving or speaking so slowly that other people could notice	D8: Motor	0.294	0.637	21.74	0.409
Thinking that you would be better off dead, or of hurting yourself in some way	D9: Suicidality ideation	0.309	0.679	21.46	0.475

*D1-D9 are PHQ-A items on depression. SD: Standard Deviation.*

**TABLE 3:** Abbreviation, Mean, Standard Deviation, and Presence of ICAST-CH abuse Events.

Abuse events	Abbreviation	Mean	SD	%Presence	Predictability
Frightened by adults’ using drugs	A1: Drug	0.500	1.130	23.39	0.207
Adults shouting in frightening way	A2: Shouting	0.954	1.293	49.35	0.368
Witnessing adults in home hitting, kicking, slapping	A3: Hit, kick, slap	0.347	0.816	21.27	0.378
Witnessing adults in home using weapons	A4: Weapons	0.134	0.537	8.36	0.319
Someone close got killed near home	A5: Killing	0.036	0.274	2.55	0.200
Having seen people being shot or rioting	A6: Shotting	0.113	0.478	7.37	0.174
Something stolen from home	A7: Stealing	0.219	0.570	16.91	0.102
Screaming	A8: Screaming	1.705	1.845	62.63	0.447
Insulted	A9: Insulted	1.430	1.924	48.6	0.393
Feeling embarrassed	A10: Embarrassed	1.169	1.561	48.16	0.459
Wished you were dead	A11: Death wish	0.514	1.142	23.48	0.520
Threatened to abandon	A12: Abandon	0.351	0.940	17.37	0.479
Locked out of home	A13: Locked out	0.279	0.697	18.82	0.311
Bullied by another child at home	A14: Bully	0.511	1.146	23.7	0.232
Being hungry or thirsty	A15: Hungry	0.070	0.421	3.8	0.260
Inadequate clothing	A16: Clothes	0.115	0.595	4.96	0.197
Unmet medical need	A17: Medical need	0.163	0.646	8.08	0.328
Feeling not cared for	A18: Not cared	0.404	1.070	17.13	0.533
Feeling unimportant	A19: Unimportant	0.675	1.398	26.08	0.608
Inadequate support/help	A20: Helpless	0.752	1.443	29.32	0.447
Threatened to hurt or kill you	A21: Hurt or kill	0.114	0.567	5.66	0.286
Pushed, grabbed, kicked	A22: Pushed	0.603	1.120	31.98	0.511
Hit, beat, spanked with hand	A23: Hit with hand	0.854	1.204	47.67	0.476
Hit, beat, spanked with object	A24: Hit with object	0.734	1.084	44.84	0.496
Trying to choke, smother, or drown	A25: Choke	0.066	0.389	3.99	0.267
Burned or scalded	A26: Burned	0.019	0.203	1.23	0.288
Locked in small place	A27: Locked	0.086	0.449	5.18	0.236
Pulled hair, pinched, twisted ear	A28: Hair pulling	0.562	1.086	30.12	0.478
Holding heavy load as punishment	A29: Heavy load	0.167	0.646	9.02	0.194
Threatened with a knife or stick	A30: Knife threat	0.100	0.494	5.75	0.301

*A1-A30 are items on abuse. SD, Standard Deviation.*

### Network Structure

**FIGURE 2:**
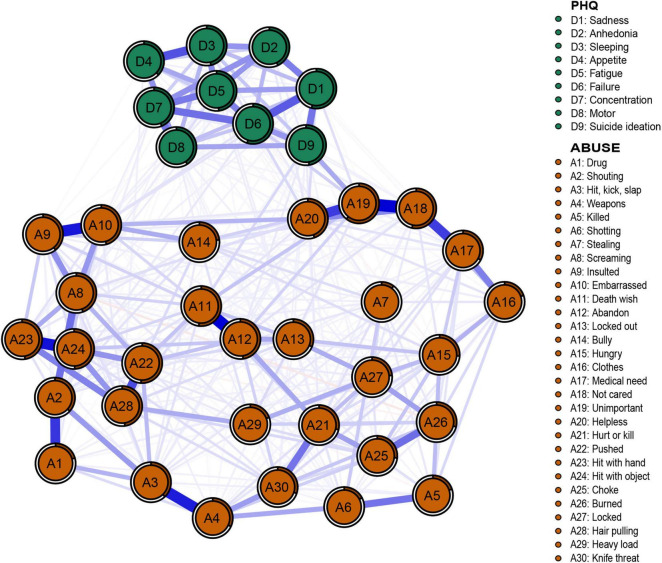
Network structure of abuse events and depression symptoms in adolescents.

The networks of depression and abuse events are shown in Figure 2. Of the 741 edges with possible connections, 428 were not zero. We found several closely connected edges in the network. The most closely linked edges between abuse events are A11 (death wish)–A12 (abandon) (weight = 0.32), A18 (not care)–A19 (unimportant) (weight = 0.32), A23 (hit with hand)–A24 (hit with object) (weight = 0.30), A9 (induced)–A10 (embarrassed) (weight = 0.29), and A3 (hit, kick, slap)–A4 (weapons) (weight = 0.29). The most closely linked edges in depressive symptoms were D3 (sleeping)–D4 (appetite) (weight = 0.22) and D1 (safety)–D6 (failure) (weight = 0.21). Predictability is shown in Figure 2 through a ring around the nodes. The predictability of all nodes ranged from 0.1 to 0.61.

### Stability and Centrality of Network

**FIGURE 3:**
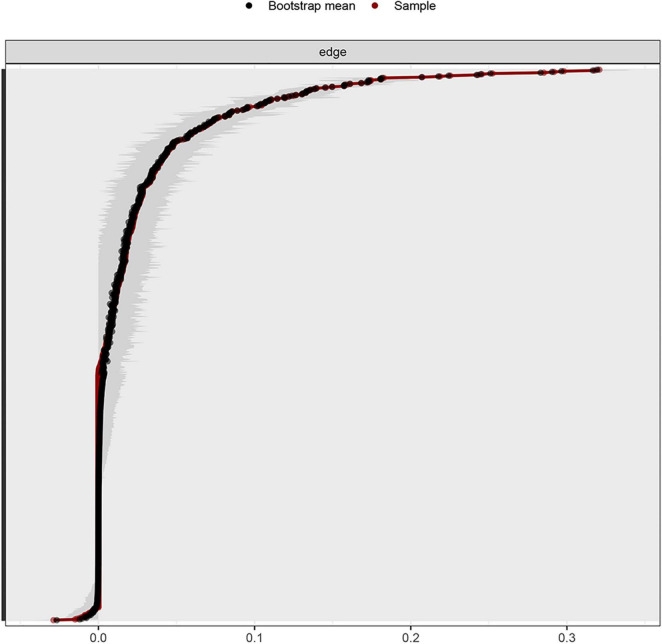
Non-parametric bootstrapped CIs of estimated edges for abuse events and depression symptoms.

Considering that the current network had 13,754 participants and the bootstrapped 95% CI was relatively narrow, the estimation of the edge weight was accurate (Figure 3). The coefficient of stability of the expected influence centrality index of the network was 0.75, higher than the recommended critical value of 0.25 (43).

**FIGURE 4:**
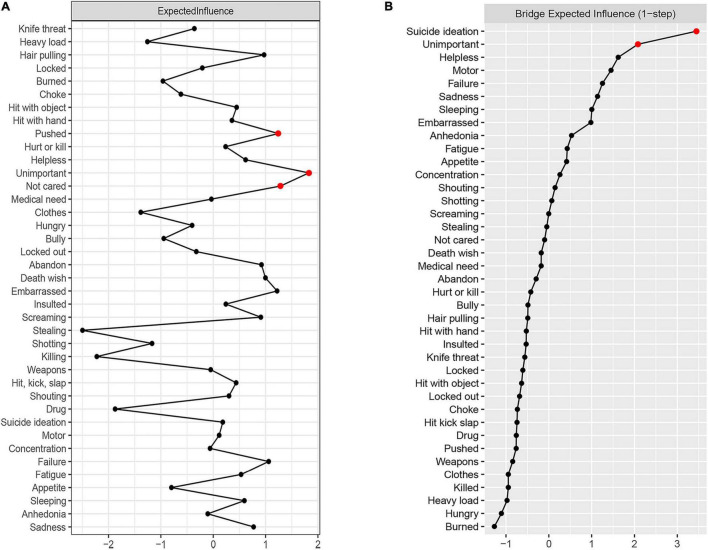
Centrality plot of the expected influence **(A)** and bridge expected influence **(B)** of abuse events and depression symptoms in the network (z-score).

The expected influence of depression and abuse networks is shown in Figure 4A. Abuse events “unimportant,” “not cared,” and “pushed” have the highest expected influence, suggesting that these three nodes have the strongest connection with other nodes in the network. Abuse events “stealing” and “killing” have the lowest expected influence in the current research, indicating that they have the weakest connection with other nodes and are the least influential in the network from the perspective of statistics.

### Bridge Nodes

The results regarding bridge nodes show that the depressive symptom “suicide ideation” and abuse event “unimportant” have the highest bridge expected influence, indicating these two variables have wider and closer connections with nodes in opposite community. Thus, abuse event “unimportant” may play more central roles in activating depressive symptoms (Figure 4B). The stability coefficient of the bridge expected influence was 0.44, which means that the stability of the bridge expected influence is acceptable.

### Flow Network of Suicide Ideation

**FIGURE 5:**
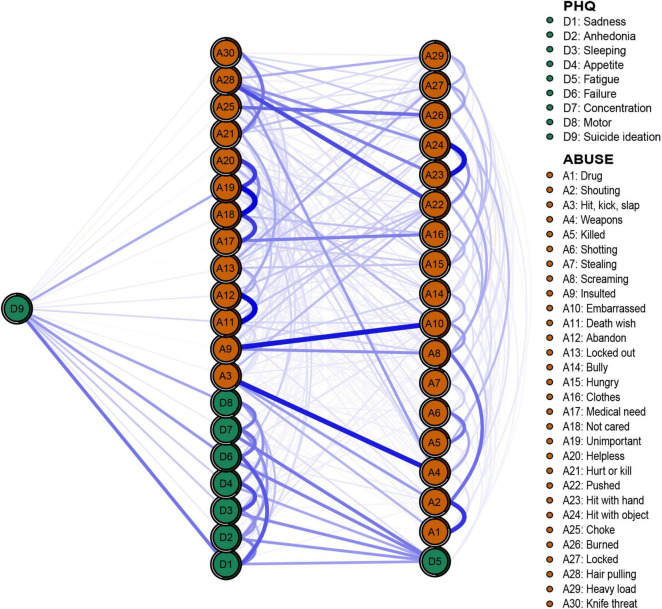
Flow network of suicide ideation.

To further explore the effect of abuse events and depressive symptoms on suicidal ideation, the flow network was constructed to reveal the association between “suicide ideation” and other nodes (Figure 5). The results showed that 20 nodes in the network were directly connected with “suicide ideation,” and 18 nodes were indirectly connected with “suicide ideation.” In addition to the depressive symptoms “safety” and “failure,” abuse event “unimportant” is strongly connected to suicidal ideation. In addition, through the predictability index, adjacent nodes can explain 47.5% of the variance of “suicide ideation.”

### Network Comparison of Suicide Ideation

Significant differences existed in the network structure of abuse events and depressive symptoms between adolescents with or without suicidal ideation, but no significant difference was found in global strength between them [network structure (M) = 0.19, *p* = 0.01; global strength (S) = 0.47, no suicidal ideation = 16.83, suicidal ideation = 17.30, *p* = 0.44]. This means that differences exist in the interaction between nodes but not in the overall connectivity of nodes in the network. There may be differences in central nodes between adolescents with or without suicidal ideation. We further analyzed the network structure of the two groups, finding that the central nodes of those with suicidal ideation were “unimportant” and “abandon,” whereas those without suicidal ideation were “pushed” and “unimportant.” The number of edges with significant differences in the two networks was 56, indicating that there were many different paths in the two networks.

## Discussion

We studied the prevalence of abuse and depression among children and adolescents in China, and the results show that the prevalence of child abuse in China is very high, especially psychological abuse. The prevalence rate is comparable with that of other regions or countries using the same tools, such as Taiwan (lifetime prevalence rate of 91% and 1-year prevalence rate of 83%) (46), India (1-year prevalence rate of 89.9%) (47), and Bangladesh (lifetime prevalence of neglect 78% to lifetime psychological abuse 97%) (48). However, the prevalence is slightly higher than that of the nine countries in the Balkan region (e.g., psychological abuse prevalence of 83.2% in Greece) (49, 50). Furthermore, the prevalence rate is higher than that measured with other tools (such as Childhood Trauma Questionnaire, according to which psychological abuse prevalence was 48.1%) (51). Therefore, future work should be focused on laying down unified criteria to facilitate the comparison of results. We also found that the prevalence of depression was also higher than previously reported in the United States (8.7–11.3%) (52) and lower than that in Ethiopia (28%) (53) and India (25%) (54). Research shows that adolescents in Confucian countries in Asia have higher levels of depression than those in European countries (55), which may be explained by a higher prevalence of abuse. Indeed, the current results show that abuses of all types are significantly positively correlated with depression. Relevant prevention policies should be formulated to reduce the prevalence of abuse in Asian countries.

The current study analyzed the characteristics of abuse event and depression networks of Chinese community adolescents. The score of each item in the questionnaire showed that depression symptoms “anhedonia” and “fatigue” and abuse event “screaming” are the most frequent symptoms. Among depressive symptoms, “fatigue” is the most frequent symptom, consistent with the study by Rice et al.; however, according to them, “anhedonia” is the most frequent depressive symptom in adults, not in adolescents (7). The difference might be explained by the fact that the samples in their study were patients with major depression, whereas those in our study were taken from community. However, common symptom also exists between these two kinds of samples, such as “fatigue.” Therefore, the differences and similarities between samples should be considered in adopting prevention and intervention measures. Among abuse events, “screaming” is the most frequent. Compared with physical abuse, verbal abuse is often underestimated. In Chinese traditional culture, shouting at children is not considered as a form of child abuse (56) but as a common way of parenting (57). Research shows that parents who suffer from verbal abuse themselves are more likely to use it against their children (58), which may explain the high frequency of verbal abuse. Therefore, education of parents is an effective way to reduce the verbal abuse. Finally, it is worth noting that the rate of “suicide ideation” in the current sample is 21.46%, much higher than that reported in other studies (12%) (36). Although the suicide rate of teenagers has gradually decreased in the past 20 years, suicidal ideation is still widespread (59), which needs further exploration.

The expected influence index of the network shows that “unimportant” is the most central node in the network of abuse events and depressive symptoms. Taking the central node as a potential target for intervention might prevent the activating and maintaining of the network of abuse events and depressive symptoms. Therefore, the intervention against “unimportant” may hopefully reduce childhood abuse and alleviate depressive symptoms among Chinese adolescents. “Unimportant” is a type of neglect, the prevalence of which varies between different cultures, which is 12.5% in India (47), 37.9% in Saudi Arabia (60), and 26.08% in the current sample, indicating that it is a common phenomenon among teenagers. In addition, the high predictability of “unimportant” (0.61) means that 61% of the variance can be explained by adjacent nodes. The nodes “not cared” and “helpless,” both belonging to neglect, have the strongest direct connection with “unimportant,” suggesting that lack of care is a serious problem among Chinese teenagers. Indeed, in China, both parents of most families need to work to support the family. In particular, some parents need to go out to work for a long time; therefore, they cannot spare enough time for taking care of their children and attending to their emotional need, leading to the development of emotional neglect symptoms of the children.

In addition, we found some edges with strong links, which exist within each community, rather than between the communities, similar to the results of previous research (28, 35, 61–64). The edges with strong links in the community of abuse events include death wish–abandon (weight = 0.32), not cared–unimportant (weight = 0.32), and hit with hand–hit with object (weight = 0.30). The strongest links in the community of depressive symptoms were sleeping–appetite (weight = 0.22) and safety–failure (weight = 0.21). Although the strongest edges do not exist across two communities, we found a very important edge between abuse events and depressive symptoms: “unimportant”–“suicide ideation” (weight = 0.11). In the current network, the edge with the strongest link in the depressive symptoms is different from that in the previous study on depression network, which found that the edges with strong links were “sad mood”–“anhedonia” or “energy”–“fatigue” (61, 62). However, our results are similar to those of Wasil et al. (65), who found that “sleep”–“appetite” is the most strongly linked edge among depressive symptoms. These differences may be explained by the fact that the first two studies adopted clinical samples (61, 62), whereas the current study and the study of Wasil et al. adopted community adolescents as samples (65). Sleep problems and weight changes are the main physical symptoms of depression in the fifth edition of the Diagnostic and Statistical Manual of Mental Disorders (DSM-5) (66), indicating that contagion of somatic symptoms may exist among community adolescents. The strongest link across two communities is “unimportant”–”suicide ideation,” which has rarely been discussed in previous studies. “Unimportant” is a type of neglect. As it has been shown that emotional neglect is associated with an increased risk of suicidal ideation (67), and this association is regulated by depressive symptoms (68), we considered that “unimportant” may arouse feelings similar to the depressive symptom “worthless,” resulting in “suicide ideation” (indirect pathways), which warrants further study in the future.

In addition, to explore the bridge nodes in the abuse event and depression symptom networks, we use the bridge expected influence as an observation index. Nodes with higher expected influence are considered to play more central roles in activating nodes from the opposite community. Thus, intervention against bridge nodes may prevent or reduce the activation from one community to the other community. We found two bridge nodes in the current network: depression symptom “suicide ideation” and abuse event “unimportant.” This indicates that intervention on “unimportant” may more effectively prevent or reduce the activation of depression symptoms.

The results of flow network show that there are 20 directly linked edges and 18 indirectly linked edges. Similar to the results of previous research (28, 36), all nodes in the network have a direct or indirect association with “suicide ideation,” supporting that “suicide ideation” is a highly complex phenomenon (69). In the current network, 48% of the variance of “suicide ideation” can be explained by adjacent nodes. The nodes directly and strongly linked to “suicide ideation” are depression symptoms “sadness” and “failure” and abuse event “unimportant.” The depressive symptoms directly associated with suicidal ideation are similar to those reported by previous studies (70), whereas the direct association between abuse event “unimportant” and suicidal ideation has not been explored previously. Previous studies show that neglect in early life can increase depression and anxiety symptoms and has a significant indirect effect on suicidal ideation (71, 72), and our results extend the finding by showing that certain types of neglect can also affect suicidal ideation directly. Our findings may provide more clues for suicide prevention and support the contribution of abuse to suicide risk. According to the three step-theory of suicide (73), suicidal ideation is the first step of suicide. Early intervention against “sadness,” “failure,” and “unimportant” may be significant in suicide prevention clinically.

Finally, we compare the differences between adolescents with or without suicidal ideation in the network of abuse events and depressive symptoms. We found significant differences in the network structure, rather than the global strength, suggesting that there are differences in the main symptoms and the interaction between the two groups with or without suicide ideation. The results showed that the central nodes in the network of those with suicidal ideation were “unimportant” and “abandon,” whereas the central nodes of those without suicidal ideation were “pushed” and “unimportant.” Therefore, “unimportant” is the most important node in the network of those with suicidal ideation, whereas its role is weakened in the network of those without suicidal ideation. This may further support that “unimportant” has an important effect on suicidal ideation. The above results might suggest different targets of intervention for adolescents with or without suicidal ideation. Therefore, it is important to clarify whether adolescents have suicidal ideation to adopt more effective intervention measures.

In summary, to reduce the prevalence of depression and suicidal ideation in adolescents, we can adopt the following measures: First, strengthening education of parents to reduce the prevalence of child and adolescent abuse. For example, the parents should be taught the appropriate parenting ways instead of abuse. Second, paying attention to the effect of neglect (e.g., abuse events “unimportant” and “not cared”) on depression of children and adolescents. To attend to the children’s emotional need, the parents need to spend more time with their children and engage in parent–child interaction (including communication) to improve parent–child relationship and alleviate depression as well as anxiety, which is highly comorbid with depression (74). Third, intervening against the depressive symptoms “sad mood,” “failure,” and abuse event “unimportant” to prevent suicidal ideation. For example, adolescents can be encouraged to participate in more physical activities, which is helpful to prevent and treat depression (75, 76). This is particularly important in China, where exam-oriented education is prevailing. Moreover, adopting targeted measures for adolescents with suicidal ideation. For example, schools can set up counseling rooms or initiate mental health activities, which can offer prevention and intervention measures for those with suicidal ideation. Studies have shown that conducting Empowering a Multimodal Pathway Toward Health Youth (EMPATHY) can effectively reduce suicidal ideation, depression and anxiety (77). The prevention of depression and suicide in children and adolescents needs policy support and the participation of more people concerned. It is necessary to develop a more comprehensive, safe, and effective long-term plan in the future.

## Limitations

Similar to other network analysis, our research has some limitations. First, this is a cross-sectional survey, which can only make undirected network estimation, but not causal inference. Second, the data are taken from community samples, which may limit the translation of the results to clinical use. Third, because the data were collected from a single region in Chongqing of China, the results of this study may not be generalizable to the adolescents in other regions. Last, the data are based on participants’ self-reports regarding specific problems; thus, the network structure may be affected by different measurement tools and time.

## Conclusion

In conclusion, to our knowledge, this study investigates the network characteristics of abuse events and depressive symptoms of adolescents in China for the first time. The results showed that the prevalence of “suicidal ideation” among Chinese adolescents is high. The abuse events “unimportant,” “not cared,” and “pushed” have the highest expected influence centrality in the network. We also found that “suicidal ideation” and “unimportant” are the bridge nodes of the network. A total of 20 nodes including “sadness,” “failure,” and “unimportant” are directly connected with suicidal ideation. There are differences in network structure between adolescents with or without suicidal ideation. Further study is needed to verify the effectiveness of intervention against these variables in the future.

## Data Availability Statement

The data analyzed in this study is subject to the following licenses/restrictions: Involving private information, the data may be available from the corresponding author for a reason. Requests to access these datasets should be directed to KL, risyaiee@msn.cn.

## Ethics Statement

The studies involving human participants were reviewed and approved by Medical Ethics Committee of the Department of Medical Psychology, Army Medical University. Written informed consent to participate in this study was provided by the participants’ legal guardian/next of kin.

## Author Contributions

KL, LL, NL, and TC: data acquisition. KL and LR: formal analysis. KL, XZ, XL, and ZF: writing. KL, LR, LZ, XZ, XL, and ZF: review and editing. All authors contributed to the article and approved the submitted version.

## Conflict of Interest

The authors declare that the research was conducted in the absence of any commercial or financial relationships that could be construed as a potential conflict of interest.

## Publisher’s Note

All claims expressed in this article are solely those of the authors and do not necessarily represent those of their affiliated organizations, or those of the publisher, the editors and the reviewers. Any product that may be evaluated in this article, or claim that may be made by its manufacturer, is not guaranteed or endorsed by the publisher.
