# Supplementary material for: The Association of Abuse and Depression With Suicidal Ideation in Chinese Adolescents: A Network Analysis
Source: Front Psychiatry. 2022 Mar 28;13:853951. doi: 10.3389/fpsyt.2022.853951 (PMC8995894; doi:10.3389/fpsyt.2022.853951)
Supplement: Supplementary file 1 [file Table_1.docx]

Supplementary Table Materia

Table S1. Intercorrelations (spearman) between depression and abuse variables

|  | Depression | Violence exposure | Psychological victimization | Neglect | Physical victimization |
| --- | --- | --- | --- | --- | --- |
| Violence exposure | 0.39** |  |  |  |  |
| Psychological victimization | 0.50** | 0.57** |  |  |  |
| Neglect | 0.52** | 0.43** | 0.59** |  |  |
| Physical victimization | 0.42** | 0.48** | 0.67** | 0.49** |  |
| Total abuse | 0.55** | 0.72** | 0.94** | 0.71** | 0.75** |

Note：**：p＜0.01 (2-tailed).
